# Optimizing Cognitive Assessment Outcome Measures for Alzheimer's Disease by Matching Wordlist Memory Test Features to Scoring Methodology

**DOI:** 10.3389/fdgth.2021.750549

**Published:** 2021-11-03

**Authors:** Jason R. Bock, Julie Russell, Junko Hara, Dennis Fortier

**Affiliations:** Embic Corporation, Newport Beach, CA, United States

**Keywords:** Alzheimer's disease, cognitive assessment, wordlist memory tests, cognitive modeling, review, digital cognitive biomarkers, clinical trials, latent cognitive processes

## Abstract

Cognitive assessment with wordlist memory tests is a cost-effective and non-invasive method of identifying cognitive changes due to Alzheimer's disease and measuring clinical outcomes. However, with a rising need for more precise and granular measures of cognitive changes, especially in earlier or preclinical stages of Alzheimer's disease, traditional scoring methods have failed to provide adequate accuracy and information. Well-validated and widely adopted wordlist memory tests vary in many ways, including list length, number of learning trials, order of word presentation across trials, and inclusion of semantic categories, and these differences meaningfully impact cognition. While many simple scoring methods fail to account for the information that these features provide, extensive effort has been made to develop scoring methodologies, including the use of latent models that enable capture of this information for preclinical differentiation and prediction of cognitive changes. In this perspective article, we discuss prominent wordlist memory tests in use, their features, how different scoring methods fail or successfully capture the information these features provide, and recommendations for emerging cognitive models that optimally account for wordlist memory test features. Matching the use of such scoring methods to wordlist memory tests with appropriate features is key to obtaining precise measurement of subtle cognitive changes.

## Introduction

Wordlist memory (WLM) tests are the most common measures of verbal episodic memory used in clinical and research settings (1, 2). They are frequently used to screen individuals prior to neuroimaging or other assessments for cognitive impairment or dementia stages of Alzheimer's disease (AD) and to monitor progressive decline and treatment effects (3). AD research has recently shifted its focus from mild cognitive impairment (MCI) and moderate AD stages toward asymptomatic or preclinical AD stages, in which the cognitive changes may be very subtle and difficult to measure (4). This has prompted the research community to examine the WLM tests that they use and develop more sophisticated scoring to achieve the greatest precision of measurement (5). A wide variety of WLM tests are in use, and each of them has a distinct set of features (e.g., wordlist length, fixed vs. shuffled word-order across trials, inclusion of semantic categories) which impact the way that individuals learn and remember the words presented in them.

In patients at risk for AD, performance on a WLM test is characterized by poorer learning, more rapid forgetting, intrusion errors, and poorer recognition that reflect pathological changes in brain regions specific to memory (6–10). A WLM test's capacity to predict AD progression at the earliest stage of change can be evaluated by examining its construction, administration procedures, and scoring methods (11). All WLM tests present a list of words over a number of learning trials and subsequently ask the examinee to freely recall as many words as they can, assessing both working memory and short-term memory across immediate learning and free recall trials as well as short-term memory alone during one or more delayed free recall trial(s). Beyond this core component, WLM tests also have varying features that impact an individual's ability to encode the presented list words into memory and retrieve them (8, 9, 12, 13). These features include the number of words to learn, properties of the words (e.g., concreteness, length, frequency, context variability, valence, and arousal), the number of learning trials, whether the list is presented in a fixed or shuffled word-order across trials, whether the words belong to semantic categories or are unrelated words, the length of the delay between learning trials and delayed free recall trial(s), whether the measure includes cued recall trials, whether it includes recognition trials, and whether those recognition trials use the same words or different words from the recall trials.

Within an individual's sequence of responding to a WLM test are distinct response patterns that, when effectively analyzed, are capable of differentiating individuals in asymptomatic, or preclinical, stages of AD from cognitively normal individuals (14). To achieve this, researchers must move away from simple summary scores (number of words recalled on a trial or in a test) and even composite scores, and toward more sophisticated methods of scoring, such as modeling of latent variables (15, 16). These approaches have improved WLM tests' capacity to identify subtle cognitive changes compared to traditional scoring. How well any WLM test can characterize cognitive performance jointly depends on the features of the test that produce the performance as well as how that performance is analyzed.

To the authors' knowledge, however, no widely-used scoring methodologies systematically take these features into account. Therefore, we discuss common WLM tests and their features as well as scoring methods and recommendations for appropriately matching them together to optimize measurement precision.

## Prevalent Wordlist Memory Tests and Their Features

Numerous WLM tests are in use today with varying wordlist features and respective applicability to memory performance. Some well-established and widely used WLM tests of verbal episodic memory include the California Verbal Learning Test-Second Edition [CVLT-II; (17)], the Rey Auditory-Verbal Learning Test or simply Auditory Verbal Learning Test [RAVLT or AVLT; (18, 19)], the Hopkins Verbal Learning Test-Revised [HVLT-R; (20)], the International Shopping List Test [ISLT; (21)], the Alzheimer's Disease Assessment Scale-Cognitive Subscale [ADAS-Cog, Word Recall; (22)], the Consortium to Establish a Registry for Alzheimer's Disease neuropsychological battery [CERAD, Word List Test; (23)], and the MCI Screen [MCIS; (24)]. The CVLT-II, AVLT, and HVLT-R are more often used in clinical settings as part of a larger flexible neuropsychological battery to assess cognitive function, and the word recall parts of the ADAS-Cog and CERAD are subtests of a fixed battery specifically designed to be used for the assessment of AD progression.

### Wordlist Memory Test Features

Each test has key features that provide advantages or disadvantages compared to other tests depending on the needs of the study. The CVLT-II and the AVLT are the longest of the word list tests, with the greatest number of words to learn and total trials, which can provide finer quantitative assessment of memory performance and greater sensitivity in distinguishing less impaired individuals and subtypes of memory impairment (11, 17, 19). However, the length of time to administer these tests and the cognitive demand required makes them less practical for research purposes and for assessing more impaired individuals (25, 26). The ADAS-Cog and the CERAD WLM subtests are shorter and have fewer trials, and due to their specific development for use in the assessment of AD, these are widely used in AD clinical research trials. The ISLT is also frequently used in AD clinical research trials; however, due to the use of words belonging to a single semantic category (food items found in a grocery store), proactive interference, a potentially useful marker of impairment in early AD, is reduced compared to tests with zero or more than one semantic category (27). The MCIS, adapted from the CERAD WLM test, includes additional feature-equivalent wordlists and uses computerized administration protocol and scoring software (24). Table 1 summarizes commonly used WLM tests of episodic memory.

**Table 1:** Commonly used wordlist memory tests and their features.

**Test name**	**# of words**	**Semantic categories**	**# of learning trials**	**Word order**	**List B**	**# of delayed trials**	**Time of longest delay**	**Cued trials**	**Old/new recognition trial**
CVLT-II	16	4	5	Fixed	Yes	2	20 min	Yes	Yes
AVLT	15	No	5	Fixed	Yes	2	20 min	No	Yes
HLVT-R	12	3	3	Fixed	No	1	20–25 min	No	Yes
ISLT	12	1	3	Fixed	No	1	10–15 min	No	No
ADAS-Cog Word Recall	10	No	3	Shuffled	No	1	5–8 min	No	No
CERAD Word List	10	No	3	Shuffled	No	1	10 min	No	Yes
MCIS	10	No	3	Fixed	No	1	5 min	No	Yes

### Wordlist Outcome Measures

Depending on which is used, WLM tests may provide the following outcome measures: individual item response data (i.e., specific words recalled), levels of total recall and recognition (i.e., summary scores), learning strategy use (e.g., semantic clustering, serial clustering, subjective clustering), primacy and recency effects, rate of new learning or acquisition, consistency of item recall, degree of vulnerability to proactive and retroactive interference, retention/forgetting over short and longer delays, cueing and recognition performance, discriminability and response bias, analysis of intrusion-error types, repetition errors, and analysis of false-positive types in recognition testing (28, 29).

### Wordlist Measures of Recognition

The addition of a recognition trial on some WLM tests helps to differentiate individuals with suspected retrieval problems, who may score better on a recognition trial than on a delayed free recall trial (28). Patients with AD have reduced benefit from cueing on a WLM recognition task due to impaired ability to consolidate learned words (8). In addition, Clark et al. (30) found that patients with amnestic MCI had poorer recognition memory abilities on a WLM recognition task compared to healthy controls, specifically driven by an increase in false-positive errors rather than a reduced number of correct responses. Their findings suggested that individuals with amnestic MCI are more sensitive to proactive interference than cognitively normal older adults. In addition, healthy older adults who took a WLM test and later developed MCI exhibited rapid decay of words 8 years prior to diagnosis, with worse recognition discriminability and a greater number of intrusion errors evident 2 years prior to diagnosis (10). In another study, investigating the relationship between WLM test performance and brain activity in cognitively normal individuals with the apolipoprotein E4 allele (a genetic risk factor for AD), compared to those without, Matura et al. (31) found that individuals with the E4 genotype showed comparatively impaired verbal recognition and cued recall memory on WLM tests. They also found a different resting state in the brain connectivity pattern between E4 carriers and non-carriers, with positive correlations between recognition discriminability scores and resting-state values in the left hemisphere of the brain associated with verbal episodic memory, suggesting a possible compensatory process occurring in this region. These study findings highlight the importance of quantifying cognitive processes, such as recognition discriminability, on WLM tests that feature a recognition component, in order to identify patterns of performance that indicate the presence of AD.

### Wordlist Measures of Serial Position

Examining serial position recall accuracy on WLM tests can also provide important information about individual differences in episodic verbal memory performance that can reveal deficits in memory encoding. The serial position effect, in which more words are recalled at the beginning (primacy) and end (recency) of a list than in the middle, is frequently analyzed in AD research (32). Patients with very mild to moderate AD exhibit a reduced primacy effect and a normal or increased recency effect (13, 33–35). Bruno et al. (36, 37) found that the ratio between immediate and delayed performance scores at the end of the list, is a sensitive marker of early MCI, with higher ratios suggesting greater risk for neurodegenerative pathology. Additionally, Tomadesso et al. (38) evaluated serial position effects in individuals with MCI who were positive for β-amyloid, a biomarker of AD, compared them to β-amyloid negative groups, and found that the β-amyloid positive group exhibited worse primacy performance. A WLM measure's presentation of words in a fixed or shuffled order across learning trials will impact the serial position effect and its capacity to inform analyses. Fixed word-order presentation maintains and reinforces serial position effects across WLM test trials, while shuffled word-order presentation eliminates the per-trial serial position effects across trials.

## Analytic Approaches

While the literature examining WLM cognitive processes shows the meaningfulness of these more sensitive measures of performance in detecting and predicting underlying memory deficits, many AD studies and clinical trials continue to use summary or memory composite scores with a set cutoff that may be disproportionally impacted by poor performance in one area (39–41). This approach dilutes a specific impairment or treatment response and leads to inefficiencies throughout a clinical trial, from screening failures to response failures that may lead to premature discontinuation of a valuable treatment, as was seen in recent AD clinical trials (16, 42).

### Composite Scoring Approaches

To overcome the limitations of summary scores in assessing early or preclinical AD, researchers developed composite scores that combine information from across multiple WLM and other tests (5). An early composite score, the ADNI-Mem, incorporates several tests used in the longitudinal Alzheimer's Disease Neuroimaging Initiative (ADNI), including the AVLT and ADAS-Cog WLM tests, and performed similarly or “slightly better” than its constituent tests (43). Wang et al. (44) developed ADCOMS, including the ADAS-Cog WLM test, and better measured clinical progression in AD and MCI than constituent tests. However, these and other composite scoring methods have not consistently demonstrated the ability to distinguish preclinical AD from normal individuals (16). More precise methods are required.

Some statistical modeling approaches go deep rather than wide. The multivariate approach uses various features of a given WLM test to analyze cognitive processes underlying learning and memory. Thomas et al. (45) used a Cox proportional hazards model to examine the added predictive value of individual cognitive processing variables (i.e., intrusion errors, learning slope, proactive interference, and retroactive interference) on a WLM test that included an interference word list. They found that intrusion errors contributed unique value in predicting progression from cognitively normal to MCI within 5 years. Another scoring model was developed for use with the MCIS, using an approach based on correspondence analysis of item response data and demographic covariates (24). This method is able to differentiate cognitively normal individuals from those with MCI with 97.3–99% accuracy (46, 47). These approaches demonstrate the value in using item-specific data from tests with complex features.

### Latent Modeling Approach

Due to high screen failure rates for β-amyloid PET when using traditional WLM test cutoff composite scores, a practical and sensitive WLM measure should be combined with a complex processing model to provide the greatest predictive capabilities. In comparison to the composite scoring approach, the latent modeling approach uses data captured by various features of WLM tests to analyze cognitive processes underlying test performance. In a simulation study, Proust-Lima et al. (15) compared inferences made by these two approaches and found that composite score risk factor accuracy is significantly reduced when constituent tests are not highly reliable or when there is systematically missing data, common in studies. In those cases, they recommend latent models.

**Figure 1:**
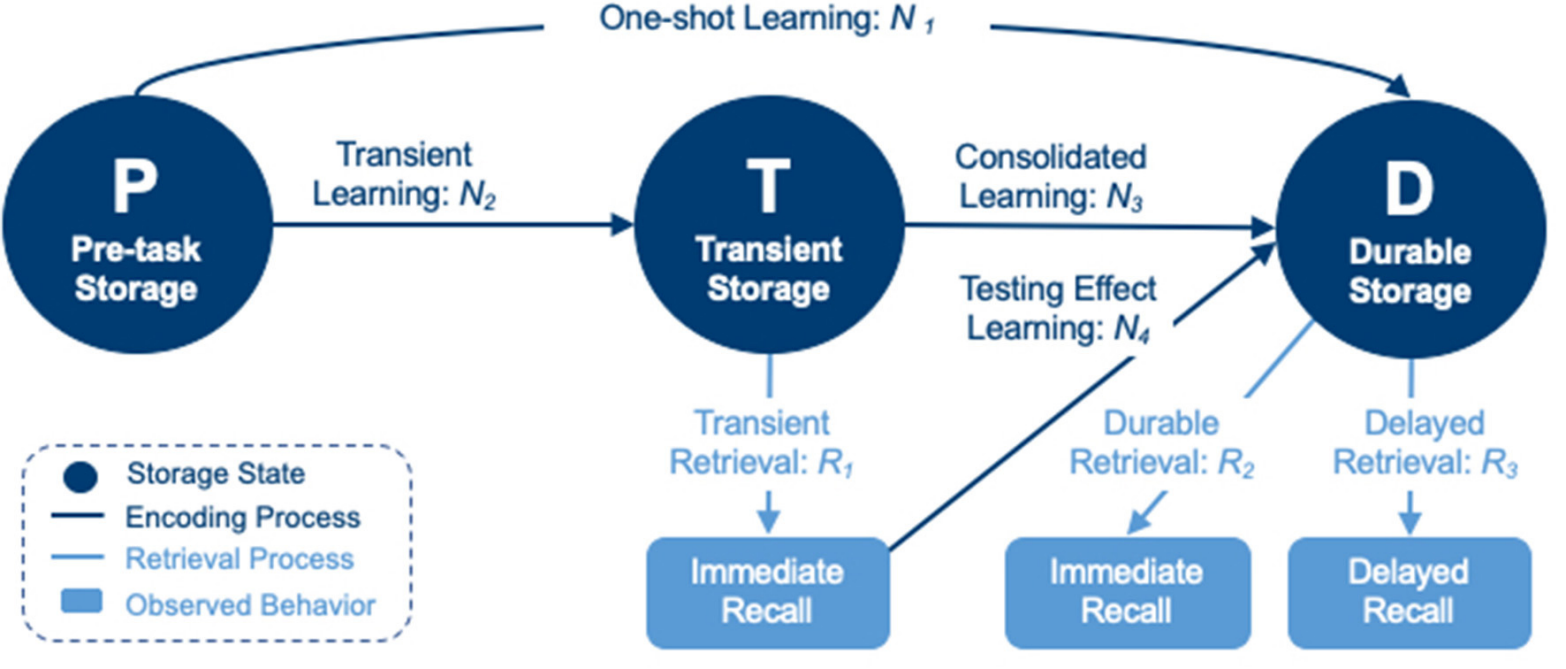
A hierarchical Bayesian cognitive processing model consisting of pre-task (P), transient (T), and durable (D) storage states, connected by encoding processes (N_1_–N_4_) and retrieval processes (R_1_–R_3_) into/from states T and D.

One such model uses multinomial processing trees and hierarchical Bayesian computational methods to quantify encoding and retrieval processes of learning in multi-trial WLM tests (48). Using this hierarchical Bayesian cognitive processing (HBCP) model (Figure 1) in a recent wordlist study, researchers were able to generate digital cognitive biomarkers (DCB) for various encoding and retrieval processes that cannot be directly observed or measured (49). These DCBs demonstrated the ability to distinguish groups of individuals with impending cognitive decline from those who would remain cognitively normal (14).

This class of model characterizes latent processes of information encoding and retrieval by utilizing item response data directly, and by building the effects of specific features that impact learning and memory directly into the model. Lee et al. (49) performed a nested analysis of a model that compared estimation of DCBs for each independent word against a model that calculated word-level DCBs from hierarchical estimations of primacy and recency directly, quantifying these features for comparison between impaired and non-impaired patients. However, this specific model relies on data that comes from a fixed word-order WLM test, as shuffled word-order WLM tests fail to produce a serial position effect. Similarly, such a model that incorporates recognition item responses would be able to quantify individuals' discriminability, and simultaneously model it with account of other cognitive processing parameters, when a WLM test includes recognition task data.

## Discussion

There is a great variety of ways to implement WLM tests as well as ways to score them. It is imperative for the study of AD, as well as memory research in general, that the lessons learned from evaluating these tests over recent decades are put into practice. Regardless of the test and the features therein, summary scores are insufficient for detection of the subtle cognitive differences in early or preclinical AD (42). Composite scores are more informative by virtue of adding the information of multiple summary scores together, but these do not take into account the unique benefits of individual WLM test features (16, 43, 44). Nevertheless, WLM tests which remove effects (e.g., shuffled word-order or control for semantic similarity) are best scored with methods that do not or cannot account for those effects. This is because greater or lesser performance for specific words of a list will produce increased error variance in methods not accounting for them, while removing these effects removes the error. However, there is valuable information in these performance differences, when a scoring method is able to account for them (32, 36, 37, 49). Expanding the scope of the data obtained from WLM tests through the use of more comprehensive analyses, such as with the described HBCP model, can significantly improve the efficiency of large-scale dementia research studies and provide valuable information about the efficacy of treatments, when paired with WLM tests that contain information produced by complex processes. While this approach has a limitation of increased complexity for interpretation and explanation of outcome measures, requiring sophistication in presenting results in clinical trials and healthcare settings, it greatly improves precision and granularity of information, compared to traditional approaches. This can be compared to machine learning, another sophisticated approach, which offers the greatest predictive capability but with even greater limitation in terms of interpretability (50). In all cases, matching the appropriate analytical method to the type of wordlist features in a given test will extract the greatest amount of information about performance and best illuminate patterns that both characterize cognitive deficits and predict cognitive change.

## Conclusion

Wordlist memory tests are commonly used for cognitive assessment, particularly in Alzheimer's disease research and screening. Commonly used tests employ a variety of inherent features, such as list length, number of learning trials, order of presentation across trials, and inclusion of semantic categories. Historically, scoring methods, such as summary scores and more recently composite scoring, have not effectively addressed differences among these features, nor have they accounted for the manner in which they may modify learning and memory during task performance. Recent developments in latent modeling have shown great potential for using specific task features to accurately quantify the underlying cognitive processes used in learning and memory. Therefore, it is beneficial to match the features of a wordlist memory test to the appropriate scoring method that accounts for those particular features. Doing so facilitates the most precise characterization of cognitive performance and optimizes the likelihood of quantifying subtle but significant cognitive changes.

## Data Availability Statement

The original contributions presented in the study are included in the article/supplementary material, further inquiries can be directed to the corresponding author/s.

## Author Contributions

JB, JH, and DF conceived and coordinated the writing of this article. JR wrote the first draft and identified references. All authors equally contributed to editing, revision, and approval of this article.

## Conflict of Interest

JB, JH, and DF are employees of Embic Corporation. JR is a consultant to Embic Corporation.

## Publisher's Note

All claims expressed in this article are solely those of the authors and do not necessarily represent those of their affiliated organizations, or those of the publisher, the editors and the reviewers. Any product that may be evaluated in this article, or claim that may be made by its manufacturer, is not guaranteed or endorsed by the publisher.
